# Osteocalcin carboxylation/undercarboxylation levels and gene variants associated with type 2 diabetes mellitus in the Chinese Han population: Osteocalcin carboxylation status and gene variants in T2DM

**DOI:** 10.3724/abbs.2025060

**Published:** 2025-04-30

**Authors:** Luyue Qi, Hong Wu, Xiangqi Li, Yang Xu, Liangyong Liu

**Affiliations:** 1 Department of Endocrinology and Metabolism the Second Affiliated Hospital of Soochow University Suzhou 215004 China; 2 Department of Endocrinology Punan Branch of Renji Hospital Shanghai Jiao Tong University School of Medicine (Punan Hospital in Pudong New District Shanghai) Shanghai 200125 China; 3 Department of Endocrinology and Metabolism Gongli Hospital of Shanghai Pudong New Area Shanghai University of Medicine & Health Sciences Shanghai 200135 China; 4 Shanghai Clinical Research Center of Bone Diseases Department of Osteoporosis and Bone Diseases Shanghai Sixth People’s Hospital Affiliated with Shanghai Jiao Tong University School of Medicine Shanghai 200233 China

Type 2 diabetes mellitus (T2DM) is an endocrine metabolic disorder characterized by insulin secretion dysfunction and/or insulin resistance. Osteocalcin (OC), or bone γ-carboxyglutamic acid protein (BGP), which was initially identified in chicken bone in 1975 [1], is a bone matrix protein predominantly produced by osteoblasts [ 2, 3]. Vitamin K-dependent carboxylation converts OC into gamma-carboxyglutamic acid (Gla)-rich carboxylated osteocalcin (cOC), which binds to hydroxyapatite and can be decarboxylated to undercarboxylated osteocalcin (ucOC) under acidic conditions. While cOC influences bone formation and mineralization, ucOC regulates energy metabolism and has garnered research interest [4]. OC enhances adiponectin production and insulin sensitivity; however, its relationship with diabetes incidence and glucose levels remains controversial [5]. This study aimed to explore the associations between T2DM, serum OC levels (including cOC and ucOC), and *OC* gene polymorphisms in the Chinese Han population. 

T2DM patients and a healthy cohort, all of Han ethnicity, were categorized into a T2DM group ( *n* = 456) and a control group ( *n* = 224). Standard clinical procedures were followed for sample collection and measurements. Serum levels of cOC and ucOC were determined via an enzyme-linked immunosorbent assay kit (TaKaRa, Dalian, China). Insulin sensitivity was assessed via homeostatic model assessment of insulin resistance (HOMA-IR). HOMA-IR = fasting blood glucose (mM) × fasting insulin (μU/mL) / 22.5. Pancreatic β-cell function was evaluated with the homeostatic model assessment of β-cell function (HOMA-β). HOMA-β = 20 × fasting insulin (μU/mL) / [fasting blood glucose (mM)–3.5]. SNPs in the *OC* gene were selected from the International HapMap Project database on the basis of specified criteria [6], totaling nine sites: rs12563631, rs2241106, rs2277872, rs2758605, rs1543294, rs1800247, rs2842880, rs759330, and rs933489. Genotyping was conducted via the SNaPshot technique. Pearson correlation and multiple linear regression analyses were performed to assess indicator correlations. Data management and statistical analyses were conducted with SPSS 24.0 software, with *P* < 0.05 indicating statistical significance. The detailed experimental procedures are provided in the Supplementary Methods. 

The detailed demographic and biochemical profiles of the study subjects are shown in Supplementary Table S1. Compared with the controls, the T2DM patients presented significantly lower levels of ucOC (0.82 ng/mL), cOC (12.19 ng/mL), and ucOC/cOC ratio (0.07) (1.17 ng/mL, 14.47 ng/mL, and 0.09, respectively) ( Figure 1A). Additionally, T2DM subjects had elevated body mass index (BMI; 25.06 kg/m ^2^), glycated haemoglobin (HbA1c; 8.95%), HOMA-IR (0.61), alkaline phosphatase (ALP; 79.00 U/L), triglyceride (TG; 1.50 mM), high-density lipoprotein (HDL; 2.58 mM), and low-density lipoprotein (LDL; 2.58 mM) levels ( *P* < 0.05), with decreased HOMA-β (1.82), alanine aminotransferase (ALT; 16 U/L), aspartate aminotransferase (AST; 17.75 U/L), high-sensitivity C-reactive protein (hsCRP; 1.68 μg/mL), and free fatty acid (FFA; 428.50 mM) levels ( *P* < 0.05). In terms of bone metabolism, T2DM patients presented increased blood phosphorus (P; 1.12 mM), carboxyterminal cross-linked telopeptide of type I collagen (ICTP; 0.78 ng/mL), procollagen type I N-terminal propeptide (P1NP; 38.30 ng/mL), and 25(OH)D levels (16.61 μg/L) ( *P* < 0.05) and decreased blood calcium, N-terminal midfragment of osteocalcin (N-MID; 11.00 ng/ml), parathyroid hormone (PTH; 31.80 ng/mL), and β-CrossLaps of type I collagen-containing cross-linked C-telopeptide (β-CTX; 0.32 ng/mL) levels ( *P* < 0.05) compared with controls.

**Figure 1:**
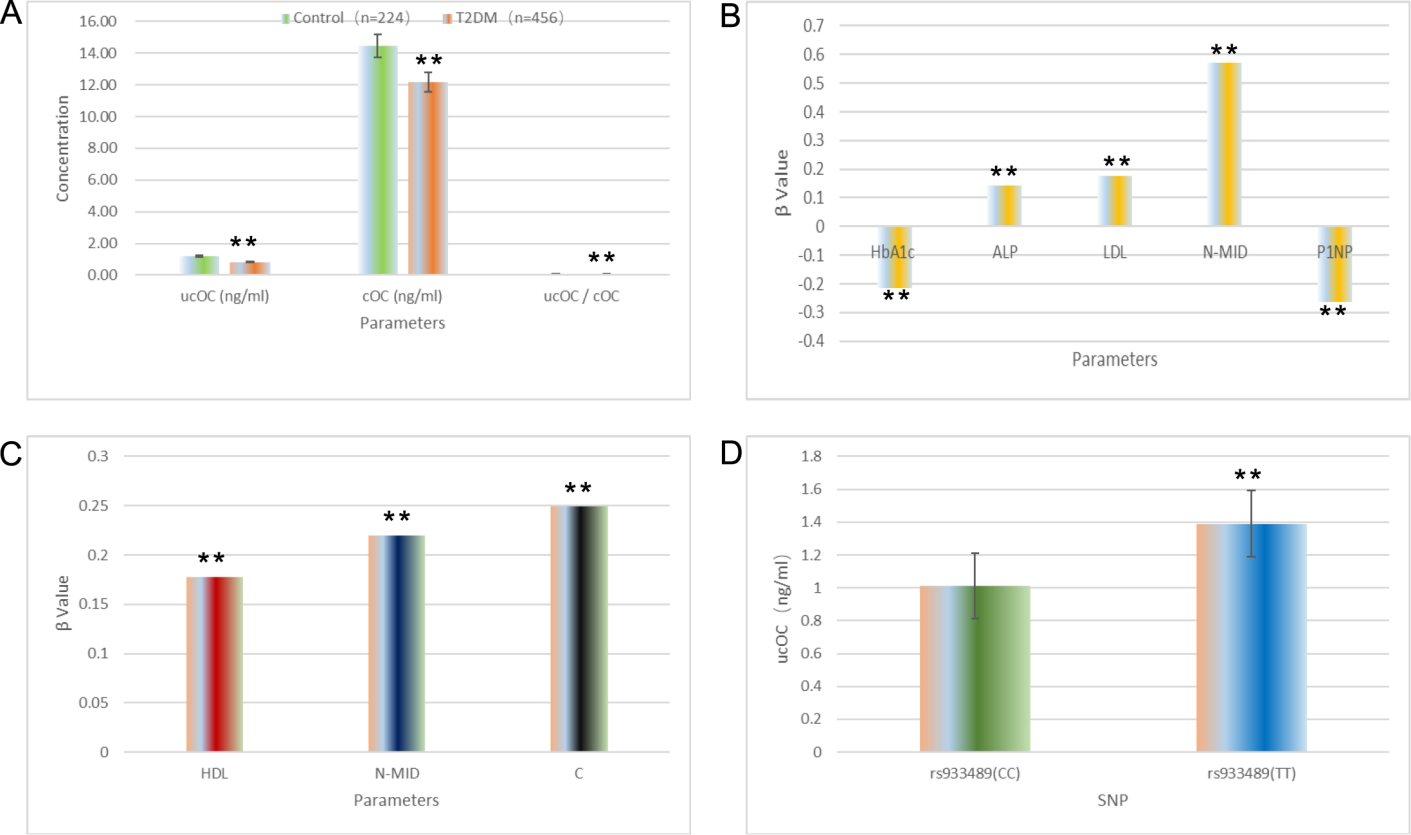
Figure 1 Osteocalcin serum protein carboxylation/undercarboxylation and gene variants in type 2 diabetes (A) T2DM patients exhibit significantly lower levels of ucOC and cOC and a significantly lower ucOC/cOC ratio. (B) cOC levels are inversely correlated with HbA1c and P1NP levels and positively correlated with ALP, LDL, and N-MID levels in the T2DM group. (C) cOC levels are positively correlated with HDL, N-MID, and PINP levels in the control group. (D) Association between ucOC levels and SNPs in the OC gene in the T2DM group. **P < 0.01.

The associations between the serum levels of cOC and ucOC and various factors were subsequently analysed ( Supplementary Tables S2 and S3). In the T2DM group, the cOC level was inversely correlated with the HbA1c and P1NP levels ( *P* < 0.05) and positively correlated with the ALP, LDL, and N-MID levels ( *P* < 0.05; Figure 1B). The ucOC level was positively associated with the N-MID level ( *P* < 0.05). In contrast, in the control group, the cOC level was positively correlated with the HDL, N-MID, and PINP levels ( *P* < 0.05; Figure 1C), while the ucOC level was correlated with the PINP level ( *P* < 0.05). 

We subsequently examined SNPs in the *OC* gene ( Supplementary Table S4) and their relationships with serum cOC and ucOC levels ( Supplementary Table S5). After adjustment for age, BMI, HbA1c, and other metabolic markers, a significant difference in the ucOC levels was observed between the CC and CT genotypes at the rs933489 locus in T2DM patients ( *P* < 0.05; Figure 1D), with the CC genotype having lower ucOC levels (1.01 ± 0.74 ng/mL). 

Our study aligns with the ground-breaking work of Lee *et al*. [4], which established OC as a regulator of glucose and lipid metabolism, thus positioning bone as an endocrine organ. Consistent with the majority of the literature, we observed a negative correlation between cOC levels and HbA1c levels in T2DM patients. However, we did not find any significant associations between OC levels and insulin resistance (HOMA-IR) or β-cell function (HOMA-β), possibly because of the longer disease duration in our cohort. Unlike previous studies, we found no significant correlation between cOC or ucOC and glycometabolic indicators in the control group, which may be attributed to the influence of drug interactions and lifestyle habits on clinical outcomes. In contrast to the findings of Zhou *et al*. [7] in postmenopausal women and men, our study did not identify a link between OC levels and lipid metabolism. This discrepancy may highlight the need for gender-specific analyses in future studies. 

On the basis of the study of Dohi *et al*. [8], which associated OC gene polymorphisms with BMD in postmenopausal women, we analyzed nine single-nucleotide polymorphism (SNP) loci for their impact on OC levels. We found no significant difference in cOC or ucOC levels across genotypes at the rs1800247 locus. However, at the rs933489 locus, the CC genotype was found to be associated with significantly lower ucOC levels than the CT genotype, a finding that warrants further investigation in larger cohorts. The lack of significant differences in OC levels among genotypes may reflect the predominant role of osteoclast activity in bone metabolism, which is influenced by ethnic and environmental factors, particularly in T2DM patients. 

Our findings of lower OC, cOC, and ucOC levels in T2DM patients underscore the pivotal role of OC in glucose and bone metabolism. Insulin may stimulate ucOC production by inhibiting forkhead box transcription factor O1 (FoxO1) and enhancing osteoclastogenesis [9]. The interplay between bone and glucose metabolism underscores the endocrine function of bone, offering novel perspectives on the regulation of systemic energy metabolism. Further research is essential to elucidate the nexus between bone metabolism and systemic energy metabolism, which may lead to the development of early diagnostic and therapeutic strategies for conditions such as diabetes and osteoporosis. 

In summary, our study leveraging clinical data from the Chinese Han population revealed significantly lower serum levels of OC, cOC, and ucOC in patients with T2DM than in healthy controls. Notably, cOC levels exhibit a significant inverse correlation with HbA1c, whereas no such correlation was observed for ucOC. Additionally, common genetic variations within the *OC* gene, specifically the CC genotype at rs933489, are associated with lower ucOC levels in Chinese Han T2DM patients. These findings provide novel perspectives on the role of OC in T2DM pathophysiology. 

The data that support the findings of this study are available upon request from the corresponding author upon reasonable request.

## Supporting information

25050Supplementary_Tables

25050Supplentary_Methods

## Supplementary Data

Supplementary data is available at *Acta Biochimica et Biophysica Sinica* online.
