# Supplementary material for: Osteocalcin carboxylation/undercarboxylation levels and gene variants associated with type 2 diabetes mellitus in the Chinese Han population: Osteocalcin carboxylation status and gene variants in T2DM
Source: Acta Biochim Biophys Sin (Shanghai). 2025 Apr 30;57(11):1901–3. doi: 10.3724/abbs.2025060 (PMC12666668; doi:10.3724/abbs.2025060)
Supplement: 25050Supplementary_Tables [file 25050Supplementary_Tables.docx]

**Supplementary Table S1. Baseline characteristics of the subjects**

| Parameters | T2DM (*n* = 456) | Control (*n* = 224) | *P* value |
| --- | --- | --- | --- |
| Age (years) | 67 (60−79) | 66 (57−77.75) | 0.248 |
| BMI (kg/m^2^) | 25.06 (22.68−27.64) | 21.79 (20.19−23.62) | <0.01 |
| Waistline (cm) | 89 (84−96) | 75 (69−78) | <0.01 |
| Course of DM (year) | 10 (3−17) | 0 | <0.01 |
| HbA1c (%) | 8.95 (7.50−10.80) | 5.00 (4.40−5.48) | <0.01 |
| HOMA-IR | 0.61 (0.41−0.88) | 0.55 (0.30−1.55) | 0.198 |
| HOMA-β | 1.82 (1.54−2.16) | 2.12 (1.85−2.33) | <0.01 |
| ALT (U/L) | 16.00 (11.80−22.95) | 20.00 (12.73−33.75) | <0.01 |
| AST (U/L) | 17.75 (14.80−24.10) | 21.00 (18.00−28.00) | <0.01 |
| ALP (U/L) | 79.00(65.25-96.75) | 72.50(59.00-88.00) | <0.01 |
| hCRP (μg/mL) | 1.68 (0.50−6.69) | 4.82 (1.22−7.84) | <0.01 |
| TC (mM) | 4.41 (3.61−5.19) | 4.51 (3.91−5.08) | 0.198 |
| TG (mM) | 1.50 (1.05−2.31) | 1.21 (0.85−1.82) | <0.01 |
| HDL (mM) | 2.58 (1.94−3.41) | 1.17 (0.88−1.52) | <0.01 |
| LDL (mM) | 2.58 (1.94−3.41) | 2.36 (1.75−2.86) | <0.01 |
| FFA (mM) | 428.50 (317.00−572.00) | 542 (388.50−683.50) | <0.01 |
| Ca (mM) | 2.19 (2.08−2.28) | 2.22 (2.12−2.32) | <0.01 |
| P (mM) | 1.12 (0.98−1.25) | 1.03 (0.89−1.14) | <0.01 |
| ICTP (ng/mL) | 0.78 (0.37−1.45) | 0.54 (0.32−0.94) | <0.01 |
| N-MID (ng/mL) | 11.00 (8.00−14.00) | 20.09 (14.00−27.45) | <0.01 |
| PTH (ng/mL) | 31.80 (23.83−42.70) | 42.89(32.55−56.16) | <0.01 |
| β-CTX (ng/mL) | 0.32 (0.21−0.48) | 0.39 (0.29−0.50) | <0.01 |
| P1NP (ng/mL) | 38.30 (28.62−54.68) | 27.16 (21.39−42.44) | <0.01 |
| 25(OH)D (μg/L) | 16.61 (12.00−21.56) | 10.31 (5.83−19.21) | <0.01 |
| ucOC (ng/mL) | 0.82 (0.50−1.49) | 1.17 (0.71−2.12) | <0.01 |
| cOC (ng/mL) | 12.19 (9.42−16.36) | 14.47 (9.60−20.41) | <0.01 |
| ucOC/cOC | 0.07 (0.04−0.12) | 0.09 (0.05−0.15) | <0.01 |

Data are presented as the median with interquartile range in the parenthesis.

**Supplementary Table S2. Multiple linear regression analysis of the association between cOC and ucOC and other independent variables in the T2DM group**

|  | ucOC (ng/mL) | | cOC (ng/mL) | |
| --- | --- | --- | --- | --- |
|  | β | *P* value | β | *P* value |
| Sex | 0.017 | 0.167 | −0.035 | 0.440 |
| Age (years) | −0.029 | 0.569 | 0.008 | 0.610 |
| BMI (kg/m^2^) | 0.009 | 0.861 | −0.105 | 0.019 |
| Waistline (cm) | 0.019 | 0.708 | −0.089 | 0.109 |
| Course of DM (year) | −0.093 | 0.072 | −0.010 | 0.824 |
| HbA1c (%) | −0.019 | 0.712 | −0.214 | **<0.01** |
| HOMA-IR | 0.004 | 0.945 | −0.019 | 0.680 |
| HOMA-β | −0.071 | 0.165 | −0.076 | 0.093 |
| ALT (U/L) | −0.035 | 0.490 | −0.029 | 0.533 |
| AST (U/L) | −0.016 | 0.752 | −0.038 | 0.405 |
| ALP (U/L) | −0.005 | 0.925 | 0.144 | **<0.01** |
| hCRP (μg/mL) | 0.023 | 0.660 | −0.101 | 0.034 |
| TC (mM) | 0.020 | 0.703 | −0.033 | -0.747 |
| TG (mM) | 0.001 | 0.987 | −0.085 | 0.064 |
| HDL (mM) | 0.011 | 0.213 | 0.052 | 0.058 |
| LDL (mM) | 0.019 | 0.373 | 0.175 | **<0.01** |
| FFA (mM) | 0.007 | 0.898 | −0.004 | 0.931 |
| Ca (mM) | 0.000 | 0.998 | 0.024 | 0.610 |
| P (mM) | −0.005 | 0.916 | 0.097 | 0.038 |
| ICTP (ng/mL) | 0.023 | 0.655 | −0.086 | 0.057 |
| N-MID (ng/mL) | 0.172 | **<0.01** | 0.571 | **<0.01** |
| PTH (ng/mL) | −0.029 | 0.640 | 0.064 | 0.224 |
| β-CTX (ng/mL) | −0.015 | 0.776 | 0.009 | 0.845 |
| P1NP (ng/mL) | 0.007 | 0.945 | −0.262 | **<0.01** |

Bold numbers indicate significant *P* values (*P*< 0.05).

**Supplementary Table S3. Multiple linear regression analysis of the association between cOC and ucOC and other independent variables in the control group**

|  | UcOC (ng/mL) | | COC (ng/mL) | |
| --- | --- | --- | --- | --- |
|  | β | *P* value | β | *P* value |
| Sex | 0.175 | 0.010 | −0.003 | 0.970 |
| Age (years) | −0.096 | 0.132 | −0.154 | 0.015 |
| BMI (kg/m^2^) | 0.006 | 0.935 | −0.097 | 0.159 |
| Waistline (cm) | 0.063 | 0.321 | 0.093 | 0.138 |
| HbA1c (%) | 0.022 | 0.721 | −0.034 | 0.584 |
| HOMA-IR | 0.018 | 0.790 | −0.054 | 0.447 |
| HOMA-β | −0.063 | 0.321 | 0.034 | 0.584 |
| ALT (U/L) | −0.036 | 0.573 | −0.071 | 0.66 |
| AST (U/L) | −0.061 | 0.367 | −0.047 | 0.468 |
| ALP (U/L) | −0.130 | −0.041 | 0.065 | 0.527 |
| hCRP (μg/mL) | −0.145 | 0.031 | −0.054 | 0.425 |
| TC (mM) | −0.127 | 0.051 | 0.000 | 0.997 |
| TG (mM) | 0.067 | 0.293 | −0.121 | 0.054 |
| HDL (mM) | −0.016 | 0.808 | 0.178 | **<0.01** |
| LDL (mM) | −0.043 | 0.496 | 0.016 | 0.803 |
| FFA (mM) | −0.068 | 0.295 | −0.035 | 0.587 |
| Ca (mM) | 0.072 | 0.296 | 0.045 | 0.528 |
| P (mM) | 0.012 | 0.847 | −0.069 | 0.282 |
| ICTP (ng/mL) | −0.127 | 0.056 | −0.038 | 0.560 |
| N-MID (ng/mL) | 0.067 | 0.333 | 0.220 | **<0.01** |
| PTH (ng/mL) | −0.060 | 0.341 | 0.019 | 0.787 |
| β-CTX (ng/mL) | 0.001 | 0.992 | 0.013 | 0.865 |
| P1NP (ng/mL) | 0.354 | **<0.01** | 0.249 | **<0.01** |

Bold numbers indicate significant *P* values (*P* < 0.05).

**Supplementary Table S4. Information of the 9 SNPs in the *OC* gene**

| SNPs | Physical position | Gene Location | Allele change | HWE  *P* value | MAF in China | MAF in this study |
| --- | --- | --- | --- | --- | --- | --- |
| rs2758605 | 156200445 | intron1 | C/G | 0.933 | 0.308 | 0.332 |
| rs1543294 | 156209857 | 5'flanking | T/C | 0.701 | 0.212 | 0.235 |
| rs1800247 | 156211825 | 5'flanking | C/T | 0.690 | 0.332 | 0.287 |
| rs759330 | 156213257 | 3'flanking | G/A | 0.674 | 0.262 | 0.226 |
| rs2241106 | 156218910 | 5'flanking | G/C | 0.607 | 0.209 | 0.252 |
| rs2842880 | 156232358 | intron13 | T/C | 0.754 | 0.257 | 0.227 |
| rs933489 | 156235120 | intron12 | C/T | 0.260 | 0.425 | 0.488 |
| rs2277872 | 156246728 | intron4 | G/T | 0.699 | 0.195 | 0.218 |
| rs12563631 | 156258953 | intron3 | C/T | 0.186 | 0.216 | 0.261 |

##

**Supplementary Table S5. The association between cOC and ucOC levels and SNPs of the *OC* gene in the T2DM group**

| SNP | Genotype | Number | ucOC (ng/mL) | cOC (ng/mL) |
| --- | --- | --- | --- | --- |
| rs2758605 | CC | 53 | 1.06 ± 0.83 | 12.73 ± 5.76 |
|  | CG | 198 | 1.33 ± 1.48 | 13.63 ± 6.17 |
|  | GG | 203 | 1.23±1.24 | 13.43 ± 6.46 |
|  | *P* |  | 0.459 | 0.579 |
| rs1543294 | CC | 57 | 1.25 ± 1.53 | 13.82 ± 6.59 |
|  | CT | 187 | 1.29 ± 1.57 | 12.88 ± 5.91 |
|  | TT | 145 | 0.97 ± 0.90 | 13.15 ± 4.17 |
|  | *P* |  | 0.540 | 0.891 |
| rs1800247 | CC | 35 | 1.48 ± 1.75 | 12.80 ± 6.68 |
|  | CT | 179 | 1.20 ± 1.10 | 13.39 ± 6.23 |
|  | TT | 240 | 1.25 ± 1.39 | 13.56 ± 6.22 |
|  | *P* |  | 0.661 | 0.455 |
| rs759330 | AA | 267 | 1.21 ± 1.14 | 13.36 ± 6.21 |
|  | AG | 163 | 1.33±1.59 | 13.61 ± 6.43 |
|  | GG | 24 | 1.16 ± 0.99 | 13.15 ± 5.65 |
|  | *P* |  | 0.598 | 0.929 |
| rs2241106 | CC | 250 | 1.27 ± 1.52 | 12.85 ± 5.74 |
|  | CG | 173 | 1.25 ± 1.04 | 14.36 ± 6.95 |
|  | GG | 31 | 1.07 ± 0.81 | 12.96 ± 5.63 |
|  | *P* |  | 0.635 | 0.242 |
| rs2842880 | CC | 267 | 1.21 ± 1.14 | 13.36 ± 6.21 |
|  | CT | 163 | 1.33 ± 1.59 | 13.61 ± 6.43 |
|  | TT | 24 | 1.16 ± 0.99 | 13.15 ± 5.65 |
|  | *P* |  | 0.598 | 0.929 |
| rs933489 | CC | 103 | 1.01 ± 0.74 | 13.02 ± 5.27 |
|  | CT | 243 | 1.39 ± 1.51 | 13.83 ± 6.87 |
|  | TT | 108 | 1.18 ± 1.24 | 12.94 ± 5.59 |
|  | *P* |  | **0.018**(CC vs CT) | 0.264 |
| rs2277872 | GG | 20 | 1.01 ± 0.93 | 12.94 ± 4.02 |
|  | GT | 159 | 1.28 ± 1.60 | 12.78 ± 5.97 |
|  | TT | 275 | 1.26 ± 1.15 | 13.85 ± 6.51 |
|  | *P* |  | 0.650 | 0.712 |
| rs12563631 | CC | 36 | 1.07 ± 0.80 | 12.84 ± 5.40 |
|  | CT | 171 | 1.27 ± 1.06 | 14.33 ± 7.04 |
|  | TT | 247 | 1.26 ± 1.52 | 12.91 ± 5.71 |
|  | *P* |  | 0.579 | 0.170 |

*P* value is the covariance analysis of Age, BMI, HbA1c, ALP, HDL, LDL, N-MID, β-CTX, and PINP adjusted ucOC and cOC levels and genotypes of the *OC* gene. Bold numbers indicate significant *P* values (*P*< 0.05).

**Supplementary Table S6. Sequences of primers used for PCR**

| SNPs | Primers (F/R) (5′→3′) | Extended primers (5′→3′) |
| --- | --- | --- |
| rs1543294 | (F) GCCCTGGCTTGGAGAATCTGTT | (RC) TTCCGCGTTCGGACTGATATCTTGGCCCAAGAGAGGCAAG |
|  | (R) TCCCAGAGGACCACGACTGAAG |  |
| rs1800247 | (F) ATTGTGGCTCACCCTCCATCAC | (RC) TTCCGCGTTCGGACTGATATCTCCGTAGGCCAAACCCAAG |
|  | (R) CCAGGGCTATTTGGGGGTCAT |  |
| rs759330 | (F) TGGGCCTGTGAGTCTGTCCAGT | (FA) TGTTCGTGGGCCGGATTAGTTTGTACCTGCCTTGTTCCAGAAACCA |
|  | (R) TGCCAGTTTGCCAGCAGCTATT |  |
| rs2842880 | (F) ggagaaaGCAGCGAAACCACAA | (FP) GTGGCTTCTCTACGTCTAGCAGTAACCT |
|  | (R) ACATGGGAGGGCTGGAAGGATA |  |
| rs933489 | (F) GGGATGACAACTCTTCTACCACAAGAC | (RC) TCTCTCGGGTCAATTCGTCCTTCCGTTTGTTAAGGCTACTCTATGCAACG |
|  | (R) GGTTTGGGGCTTTCCTCCCTAC |  |
| rs2241106 | (F) CATCTCCAGGGCCTTCACTCAA | (RC) TTGGAAAGGCTTCTTTGAAGTGGAAGC |
|  | (R) GCACTGAAGATGGCATCCTGAAA |  |
| rs2758605 | (F) GCAAGGGAGAGGGAGGAAACAA | (RC) TTCAATGGCTCATTCCTCTTGGACACAC |
|  | (R) gcccagccAAGCCTGATTTTA |  |
| rs2277872 | (F) GTCCACAATCCCAGGCCTCTCT | (FP) AAATCCAAACTTCRTAAACCATTTGTATGTAGT |
|  | (R) TGACCCCCTCATAGGTCAGTGG |  |
| rs12563631 | (F) tctggtccaggcttcccatttt | (FP) gtagagtacttaaaacaatctagtacttggtgccTTT |
|  | (R) accacgcctggcaccaagta |  |
