# Supplementary material for: Osteocalcin carboxylation/undercarboxylation levels and gene variants associated with type 2 diabetes mellitus in the Chinese Han population: Osteocalcin carboxylation status and gene variants in T2DM
Source: Acta Biochim Biophys Sin (Shanghai). 2025 Apr 30;57(11):1901–3. doi: 10.3724/abbs.2025060 (PMC12666668; doi:10.3724/abbs.2025060)
Supplement: 25050Supplentary_Methods [file 25050Supplentary_Methods.docx]

**Supplementary Methods**

**Research participants**

This study enrolled patients with type 2 diabetes mellitus (T2DM) from the Department of Endocrinology at Punan Branch of Renji Hospital, Shanghai Jiao Tong University School of Medicine, (Shanghai, China), and healthy volunteers from the Physical Examination Centre of the Sixth People’s Hospital Affiliated to Shanghai Jiaotong University (Shanghai, China), between 2017 and 2018. The study protocol was approved by the Independent Ethics Committees of both hospitals. Participants were all Chinese Han descent. T2DM patients were diagnosed in accordance with the Chinese Diabetes Society (CDS) Guidelines for the Prevention and Treatment of T2DM (2017 edition). The cohort was bifurcated into two groups based on T2DM status at recruitment: 456 individuals in the T2DM group and 224 in the normal control group.

**Clinical assessments**

Participants provided data on age, diabetes duration, and fracture history. Anthropometric measurements including height and weight were taken to determine body mass index (BMI). Information on dietary habits, physical activity, and use of tobacco, alcohol, and milk was collected. A physical examination and routine laboratory assessments were performed, encompassing a complete blood count, urinalysis, blood chemistries (calcium and phosphorus), fasting glucose and insulin levels, lipid panel, C-reactive protein (CRP), and evaluations of liver and kidney functions, along with bone metabolism markers. Informed consent was obtained before data collection and sample retention.

After a 12-h fasting, urine and serum samples were obtained for analysis. The Clinical Laboratory at Shanghai Punan Hospital conducted tests for fasting blood glucose, fasting insulin, HbA1c, high-sensitivity CRP (hsCRP), phosphorus, calcium, liver and kidney function, total cholesterol (TC), triglycerides (TG), high-density lipoprotein cholesterol (HDL), low-density lipoprotein cholesterol (LDL), free fatty acids (FFA), 25-hydroxy vitamin D [25(OH)D], parathyroid hormone (PTH), Cross-linked Carboxy-terminal telopeptide of type I collagen (ICTP), N-terminal midfragment of osteocalcin (N-MID), and β-CrossLaps of type I collagen-containing cross-linked C-telopeptide (β-CTX). Enzyme-linked immunosorbent assay was performed to measure serum levels of carboxylated osteocalcin (cOC) and undercarboxylated osteocalcin (ucOC). Insulin resistance was quantified using the homeostatic model assessment (HOMA-IR). HOMA-IR = fasting blood glucose (mM) × fasting insulin (μU/mL)/22.5. Pancreatic β-cell function was assessed with the HOMA-β. HOMA-β=20 × fasting insulin (μU/mL) / (fasting blood glucose (mM)− 3.5).

**SNP selection and genotyping**

We selected candidate single nucleotide polymorphisms (SNPs) within the *OC* gene from the International HapMap Project database, with the following criteria: (1) SNPs were validated in the Chinese population; (2) Minor allele frequency (MAF) exceeded 0.05; (3) Pairwise linkage disequilibrium (LD) with an r2 value greater than 0.8; and (4) SNPs implicated in genome-wide association studies (GWAS) or confirmed in other research were included. Consequently, 9 SNPs were selected for this study: rs12563631, rs2241106, rs2277872, rs2758605, rs1543294, rs1800247, rs2842880, rs759330, and rs933489.

For genotyping, genomic DNA was extracted from peripheral venous blood samples of participants using a standard phenol-chloroform method. The selected nine SNPs were genotyped using the SNaPshot SNP genotyping technique, with primer sequences detailed in the following **Supplementary Table S6**.

**Statistical Analysis**

Continuous variables exhibiting normal distribution were presented as the mean ± standard deviation (SD), whereas those with non-normal distribution were reported as median and interquartile range (IQR). For non-normally distributed data, logarithmic transformation was applied prior to analysis. Independent samples *t*-tests and approximate *t*-tests were utilized to compare group differences after assessing variance homogeneity. Pearson correlation analysis and multiple linear regression analysis were employed to examine the relationships among indicators. Linear regression models were applied to investigate the associations between serum OC, cOC, ucOC levels, and metabolic indices, including glucose and lipid metabolism, as well as bone metabolism markers, and to assess the link between SNP genotypes and serum OC levels. Statistical data entry and analysis were conducted using SPSS 24.0 software, with *P* < 0.05 indicating statistical significance.
